# Supplementary material for: A UMLS-based spell checker for natural language processing in vaccine safety
Source: BMC Med Inform Decis Mak. 2007 Feb 12;7:3. doi: 10.1186/1472-6947-7-3 (PMC1805499; doi:10.1186/1472-6947-7-3)
Supplement: Additional file 13 — RAP application directory. Sets up directory for RAP (RDF application for PHP) source code [file 1472-6947-7-3-S13.gz › rap/doc/phpdoc/utility/FindIterator.html]

xml version="1.0" encoding="iso-8859-1"?


Docs For Class FindIterator


## Class FindIterator

Description

Description |
Vars (details)
| Methods (details)

Iterator for traversing statements matching a searchpattern.

FindIterators are returned by model->findAsIterator() Using a find iterator is significantly faster than using model->find() which returns a new result model.

History:

- 08-05-2004 : First version of this class.

- access: public
- author: Tobias Gau� <tobias.gauss@web.de>
- version: V0.9.1

Located in /util/FindIterator.php (line 25)

```
Object
   |
   --FindIterator
```

Variable Summary

Description |
Vars (details)
|
Methods (details)

mixed
$object

mixed
$predicate

Method Summary

Description |
Vars (details)
|
Methods (details)

FindIterator
FindIterator
(object MemModel &$model, object Subject $sub, object Predicate $pred, object Object $obj)

statement
current
()

boolean
hasNext
()

statement
next
()

Variables

Description |
Vars (details)
|
Methods (details)

mixed
$object
(line 55)

mixed
$predicate
(line 54)

Methods

Description |
Vars (details)
Methods (details)

Constructor FindIterator (line 67)

Constructor

- access: public

FindIterator

FindIterator
(object MemModel &$model, object Subject $sub, object Predicate $pred, object Object $obj)

- object MemModel
  &$model
- object Subject
  $sub
- object Predicate
  $pred
- object Object
  $obj

current (line 113)

Returns the current matching statement.

- return: or NULL if there is no current matching statement.
- access: public

statement

current
()

hasNext (line 81)

Returns TRUE if there are more matching statements.

- access: public

boolean

hasNext
()

next (line 95)

Returns the next matching statement.

- return: or NULL if there is no next matching statement.
- access: public

statement

next
()

#### Inherited Methods


Inherited From Object

> Object::toString()

Documentation generated on Thu, 7 Jul 2005 13:41:27 +0200 by phpDocumentor 1.3.0RC3
